# Health Beneficial Phytochemicals in *Dioscorea caucasica* Lipsky Leaves and Tubers and Their Inhibitory Effects on Physiologically Important Enzymes

**DOI:** 10.3390/plants11101341

**Published:** 2022-05-18

**Authors:** Aušra Adomėnienė, Audrius Pukalskas, Ona Ragažinskienė, Petras Rimantas Venskutonis

**Affiliations:** 1 Department of Food Science and Technology, Kaunas University of Technology, Radvilenų Pl. 19, LT-50254 Kaunas, Lithuania; ausra.adomeniene@ktu.edu (A.A.); audrius.pukalskas@ktu.lt (A.P.); 2 Kaunas Botanical Garden, Vytautas Magnus University, Žilibero 6, LT-46324 Kaunas, Lithuania; ona.ragazinskiene@vdu.lt

**Keywords:** *Dioscorea caucasica*, flavonoids, phenolic acids, α-amylase, α-glucosidase, acetylcholinesterase, angiotensin-converting enzyme

## Abstract

*Dioscorea caucasica* Lipsky is a tertiary relict endemic plant naturally growing in the western part of the trans-Caucasus regions; it has adapted and successfully grows in the temperate region of the Baltic countries. Information about its phytochemical composition and bioactivities is rather scarce. This study reports the results of the identification of 41 compounds in *D. caucasica* leaf and tuber hydroethanolic extracts using UPLC-QTOF/MS. Organic acids were found in both extracts; hydroxycinnamates and flavonoids were the main phytochemicals in the leaves, while steroidal glycosides, fatty acids (mainly hydroxylated) and carbohydrates were found in the tubers. Leaf extracts inhibited enzymes in a dose-dependent manner and were remarkably stronger inhibitors of physiologically important enzymes, namely α-amylase (48.6% at 480 µg/mL), α-glucosidase (IC_50_ = 41.99 and 47.95 µg/mL with and without 0.1 M Na_2_CO_3_), acetylcholinesterase (45.85% at 100 µg/mL) and angiotensin-converting enzyme (IC_50_ = 829.7 µg/mL), most likely due to the presence of some quantified polyphenolic antioxidants. The mode of inhibition of α-glucosidase and acetylcholinesterase was assessed via kinetic studies based on Lineweaver–Burk inhibition plots. Leaf and tuber extracts acted as mixed-type and competitive inhibitors of α-glucosidase, respectively; the leaf extract demonstrated an uncompetitive inhibition mode of acetylcholinesterase. It is expected that this new knowledge of *D. caucasica* will serve for its valorization in developing new health beneficial ingredients for functional foods and nutraceuticals.

## 1. Introduction

The interest in health beneficial natural substances has been steadily increasing during the last few decades. This tendency has been fostered by the fast developments in the area of functional foods and nutraceuticals, which have contributed in shifting healthcare concepts towards the increasing role of preventive medicine. In addition, such a generic characteristic of nutritional and healthcare products as ‘naturalness’ has become very popular among consumers [1]. Due to the vast number of plant species, which are the main sources of bioactive phytochemicals, this tendency has stable and sustainable support, particularly considering the presence of many under-investigated plants.

Plant phytochemicals exert various bioactivities, which may provide numerous health benefits, particularly in preventing and/or delaying the development of age-dependent degenerative and chronic diseases [2]. Among the mechanisms of their bioactivities, antioxidant and antimicrobial properties, molecular signaling, inhibition of enzymes and other effects have been reported for various plant preparations and purified natural compounds [3,4,5,6,7]. Many studies have reported the inhibition of physiologically important enzymes by extracts isolated from botanicals, which correlates with various health benefits [8,9,10,11]. For instance, it is well established that the inhibition of amylase and glucosidase enzymes may reduce the postprandial glycemic index, which is linked to the development of diabetes [12], while inhibition of angiotensin-converting enzyme (ACE) may assist in maintaining normal blood pressure [13].

This study is focused on the phytochemical characterization of hydroethanolic extracts of *Dioscorea caucasica* Lipsky leaves and tubers and the evaluation of their effects on the activity of physiologically important enzymes. *Dioscorea* is a large genus of the Dioscoreaceae family, comprising around 715 species, which probably originates from the South-East Asia region [14]. The yam is a common name for some edible tuber-forming *Dioscorea* species. Although the largest diversity of yams is present in the humid tropical and subtropical regions of the world, a number of *Dioscorea* spp. grow in the temperate and mountainous regions as well. All yams are perennial, herbaceous, dioecious twining climbers producing dry capsules, although, occasionally, both male and female flowers can be found on the same plant [15]. Despite the botanical similarities, the biochemical composition of *Dioscorea* species may differ significantly; therefore, raw materials of different origin have been claimed to possess diverse nutritional, economic and medicinal value. Some yams, which are cultivated in the humid tropical regions, are economically important crops as a source of cheap starchy food [16], whereas in the temperate climate regions, *Dioscorea* plants are mainly used due to their health benefits. It should be noted that the majority of *Dioscorea* spp. studies have been focused on yam tubers, whereas plant leaves may also present potential as renewable resources for various bioactive compounds [17]. 

*D. caucasica* is one of the most valuable representatives of the genus. This is a rare tertiary relict endemic plant naturally growing in the Caucasus and mainly found in the western part of the trans-Caucasus regions of Georgia (Abkhazia), Krasnodar (Adler) and some others [18]. It is widely grown in the botanical gardens of Northern Europe as well [18]. Despite the limitations in natural resources, this medicinal plant has received increasing scientific and commercial attention due to the expanding information on the properties of its bioactive compounds. *D. caucasica* is considered as a high-value native medicinal plant in the Caucasus, whose rhizomes with roots are used for producing folk medicines and health-promoting preparations in the form of ground powder, decoctions, herbal teas or ethanol extracts. Such preparations are prescribed as anti-atherosclerotic, strengthening the function of the brain and contributing to the improvement of cognitive capabilities, as well as being beneficial to the blood circulatory/cardiovascular system as a natural medicine. It has also been reported that *D. caucasica* nutraceuticals may assist in normalizing blood pressure, both in hypertensive and hypotensive patients; therefore, in Russia, *D. caucasica* rhizomes and roots are used in the pharmaceutical industry to manufacture novogalene drugs for treating atherosclerosis [19]. 

However, the majority of the ethnopharmacological health benefits of *D. caucasica* preparations are not sufficiently supported scientifically, both in terms of their bioactivities and phytochemical composition. Several bioactive compounds isolated from *D. caucasica*, especially steroidal saponins dioscin and diosgenin, have shown important pharmacological activities, such as suppressing cellular viability and inducing apoptosis in ovarian cancer cells [20], protecting against coronary heart diseases, exerting anti-inflammatory effects by mitigating oxidative stress [21,22,23] and reducing neuropathic pain [10]. The studies conducted with *Dioscorea* species have also revealed that their constituents might reduce the expression of α-amylase and α-glucosidase [7,9] and inhibit angiotensin-converting enzyme [24] and pancreatic lipase [8]. To the best of our knowledge, only one report is available on *D. caucasica* phytochemicals, particularly polyphenolic constituents: Szakiel et al. (2017) [25] reported the presence of non-glycosylated triterpenoids in the lipophilic fraction of *D. caucasica* leaves from Poland. 

Currently, as *D. caucasica* has been introduced and successfully adapted in the temperate region of the Baltic States, the interest in this plant, as a promising source of functional ingredients for foods and nutraceuticals, is increasing in the northern and other countries as well. The overall aim of this study was to screen the phytochemicals in hydroethanolic extracts of *D. caucasica* leaves and tubers and to evaluate their effects on some physiologically important enzymes, namely α-amylase, α-glucosidase, acethylcholinesterase and angiotensin-converting enzyme. It is expected that this new scientific knowledge of *D. caucasica* will be useful for its valorization in developing new health beneficial ingredients for functional foods and nutraceuticals. 

## 2. Results

### 2.1. Phytochemical Composition of Extracts 

#### 2.1.1. Identification of Secondary Metabolites Using UPLC-QTOF/MS

The compounds detected in the extracts are listed in Table 1. Several organic acids were identified in *D. caucasica* leaf and tuber extracts: quinic **1** ([M − H]^−^ ion at *m*/*z* 191.0556), malic **2** ([M − H]^−^ ion at *m*/*z* 133.0137) and citric/isocitric **4** ([M − H]^−^ ion at *m*/*z* 191.0191) acids were detected at t_R_ 0.4 and 1.2 min, respectively. Shikimic acid **3** ([M − H]^−^ ion at *m*/*z* 173.0450) with t_R_ 0.5 min was detected only in the leaf extract, while piscidic acid **5** ([M − H]^−^ ion at *m*/*z* 255.0505) with t_R_ 1.8 min was found only in the tuber extract. Shikimic acid was reported in *D. elephantipes*, *D. sylvatica* and *D. mexicana* [26], while piscidic acid was reported in *D. nipponica* tuber [27]. However, this acid was not reported previously in *D. caucasica* tubers. The MS data of 2-deoxy-2,3-dehydro-*N*-acetylneuraminic acid (Neu2en5Ac) **33** ([M − H]^−^ *m*/*z* 290.0875) at t_R_ 0.9 min perfectly matched the fragmentation pattern of this sialic acid derivative, which has been reported in various biomaterials, including plants. The identity of pyroglutamic acid **34** ([M − H]^−^ ion *m*/*z* 128.0347) at t_R_ 0.9 min and glucoheptonic acid **32** ([M − H]^−^ ion *m*/*z* 225.0610) at t_R_ 0.3 min is also in agreement with the previously reported data [28,29]. These compounds were found only in the leaf extract.

It is evident that phenolic acids and flavonoids are the most important phytochemicals in the leaf extract: based on MS and other data, eight hydroxycinnamic acid derivatives and seven flavonoids were identified (Table 1). Caffeoylquinic acid (CQA) isomers have the chemical formula of C_16_H_18_O_9_ and the monoisotopic mass of 354.0950. Similar [M − H]^−^ precursor ions with *m*/*z* 353.0872 were recorded for the compounds **6**, **10**, **12** (t_R_ = 1.5, 1.7, 1.9 min), which indicates the presence of several CQA isomers. The first compound **6** was identified as 3-caffeoylquinic acid. The second compound **10** was assigned to 5-chlorogenic acid based on its dimer *m*/*z* 707.1823 [2M − H]^−^, which yielded the peaks of *m*/*z* 353.0872 [M − H]^−^ and *m*/*z* 191.0556 [M − H]^−^. The spectra are in agreement with those previously reported [31,32] and, in addition, were confirmed with an authentic standard. Based on a precursor ion at *m*/*z* 297.0612 [M − H]^−^, the compound **9** was tentatively identified as caffeoylthreonic acid. The compounds **11, 12** produced molecular ions [M − H]^−^ with *m*/*z* 337.0923, 353.0872 and the fragments of *m*/*z* 191.0556 [M–H–146]^−^ and *m*/*z* 191.0556 [M–H–162]^−^, respectively; these spectral data enabled us to assign the peaks to coumaroylquinic and 4-caffeoylquinic acids, respectively. Compound **13** (t_R_ = 2.0 min) yielded an ion at *m*/*z* 335.0766 [M − H]^−^ and was identified as caffeoylshikimic acid [33], while the compound **14** (t_R_ = 2.1 min) yielded an ion at *m*/*z* 367.1029 [M − H]^−^ and was assigned to feruloylquinic acid. 

The compounds **15**–**21** were identified as flavonoids, all being quercetin derivatives (Table 1). The chemical formula of quercetin (3,3′,4′,5,7-pentahydroxyl-flavone) is C_15_H_10_O_7_ and the monoisotopic mass is 302.04265. In general, *D. caucasica* leaf extract flavonoids produced spectra characteristic for [M − H (3-*O*-glycoside)]^−^. Thus, in the negative ion mode, the compounds **15**, **16** with molecular ions at *m*/*z* 609.1455 and 463.0876 and retention times of 2.3, 2.4 min, correspond to quercetin 3-*O*-rutinoside (rutin) and quercetin 3-*O*-glucoside (isoquercitrin). The identity was also confirmed by the reference standards. The compound **19** (t_R_ = 2.6 min) gave *m*/*z* of 447.0927 and, based on comparison with the spectral data available in the literature [35], it was assigned to quercetin 3-*O*-rhamnoside (quercitrin). The compounds **17**, **18** (t_R_ = 2.5 min) demonstrated precursor ions [M − H]^−^ at *m*/*z* 549.0880 and *m*/*z* 505.0982, respectively. Consequently, the compounds were identified as quercetin-3-*O*-malonylglucoside and quercetin-3-*O*-acetylglucoside, respectively h [34]. The ions of the compound **20** (*m*/*z* 533.0931) and compound **21** (*m*/*z* 489.1033), corresponding to the molecular formulas C_24_H_21_O_14_ and C_23_H_21_O_12_, respectively, enabled the tentative identification of quercetin 3-*O*-malonylrhamnoside and quercetin 3-*O*-acethylrhamnoside, respectively.

Except for hexose **22** of unknown structure, other sugars were detected only in *D. caucasica* tubers. Sucrose **23** ([M − H]^−^ ion *m*/*z* 341.1083) [26,30,36] and the following compounds **24** ([2M − H]^−^ ion at *m*/*z* 683.2246) and **25** ([3M − H]^−^ ion at *m*/*z* 1025.3408) correspond to the spectral characteristics of deprotonated dimer and trimer oligosaccharide ions [37]; they are included in Table 1 as unseparated sugars.

Long-chain fatty acids and their hydroxylated derivatives are common in various plant materials, including yam tubers of *Dioscorea* species [38,43,44]. Spectral data of the compound **31**, [M − H]^−^ ion *m*/*z* 279.2324 (t_R_ = 8.8 min, fits linoleic acid (C18:2), and those of **29** [M − H]^−^ ion *m*/*z* 295.2273 (t_R_ = 6.9 and 8 min) correspond to linolenic acid (C18:3). Other derivatives suggest the structures of hydroxylated fatty acids: **30** [M − H]^−^ ion *m*/*z* 271.2273 at (t_R_) 8.4 min and **26** [M − H]^−^ ion *m*/*z* 329.2328 at (t_R_) 4.6 min correspond to hydroxyhexadecanoic and trihydroxyoctadecenoic acids, respectively. Hydroxyoctadecatrienoic acid **27** [M − H]^−^ ion *m*/*z* 293.2117 was detected only in the leaf extract at (t_R_) 5.8 min. MS data do not provide sufficient information on the exact position of the hydroxy group in the fatty acid chain. Furthermore, MS data of the compound **28**, [M − H]^−^ ion *m*/*z* 358.2593 at (t_R_) 6.8 min, suggest the molecular formula of C_19_H_36_NO_5_, which fits a fatty acid ester (https://www.lipidmaps.org accessed on 12 December 2021 [39]). The latter was assigned to a carnitine derivative, hydroxy dodecanoylcarnitine. Conjugates of fatty acids with amino acids occur widely in food from animal sources, but there is limited availability in plants. The obtained mass spectra do not allow us to identify the precise site of hydroxylation.

It was not possible to elucidate the exact structures of **36**, **37**, **38**, **39**, **40**; however, based on their [M − H]^−^ *m*/*z* of 1109.5379 (t_R_ = 3.3 min), 1093.5431(t_R_ = 3.7 min) and 1075.5325 (t_R_ = 4.3 min), 929.4746 (t_R_ = 6.5 min), 913.4797 (t_R_ = 6.6 min), which correspond to formulae of C_52_H_85_O_25_, C_52_H_85_O_24_, C_52_H_83_O_23,_ C_46_H_73_O_19_ and C_46_H_73_O_18_, respectively, most likely belong to steroidal glycosides, which are abundant in yams [41] and some other plants [40,42]. Spectral data of the compound **41** [M − H]^−^ ion *m*/*z* of 767.4217 (t_R_ = 6.8 min) also indicate steroidal saponin pentandroside B [42]. Furthermore, the compound **45** [M − H]^−^ ion *m*/*z* 723.3803 corresponding to C_34_H_59_O_16_ detected in tubers at 6.8 min and the compound **44** [M − H]^−^ ion *m*/*z* 721.3646 detected in leaves at 5.6 min, and corresponding to C_34_H_57_O_16_, most likely belong to galactolipids. The compound **35** producing [M–H]^−^ ion at *m*/*z* 345.1186 at (t_R_ = 1.4 min) fit well with the relevant data of an iridoid glycoside and was tentatively identified as an aucubin. This iridoid is commonly found in different anatomical parts of plants and is responsible for the defensive function.

#### 2.1.2. Quantitative Analysis of the Main Secondary Metabolites Using HPLC

**Figure 1:**
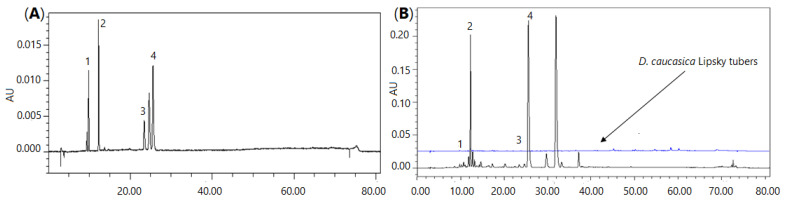
Chromatographic profile of *D. caucasica* leaf and tuber extracts (**B**) and the mixture of reference phenolic compounds (**A**): 1—neochlorogenic acid, 2—chlorogenic acid, 3—rutin, 4—isoquercitrin.

The content of neochlorogenic acid, chlorogenic acid, rutin and isoquercitrin in the *D. caucasica* leaf extract was 5.89, 116.6, 4.02 and 187.2 µg/mL, respectively. Thus, quercetin glycoside and isoquercitrin were the most abundant phenolic constituents recovered from the plant leaves. In general, these data agree with the UPLC-QTOF/MS analysis, which demonstrated the highest *m*/*z* intensities for the major quantified compounds. As may be judged from the peak area in the chromatographic profile (Figure 1), some other constituents may also be present in the extract at high concentrations. Based on the peak *m*/*z* intensities in the UPLC-QTOF/MS chromatograms, these compounds might be quinic acid, which forms esters with phenolic acids, and quercitrin, which eluted after isoquercitrin (Figure 1), i.e., similarly as in the UPLC-QTOF/MS chromatogram. The content of biologically active phenolic compounds in the leaves was remarkably higher than in the tubers. For instance, the content of neochlorogenic and chlorogenic acids in the tuber extract was only 0.687 and 1.869 µg/mL, respectively. Quantification of other tuber phytochemicals was beyond the scope of this study; however, based on the peak *m*/*z* intensities in the UPLC-QTOF/MS chromatograms, the major compounds in the tuber extract might be some sugars, piscidic acid, linolenic acid and two steroidal glycosides with t_R_ 3.7 and 6.6 min (Table 1).

### 2.2. Enzyme Inhibitory Properties of D. caucasica Extracts

#### 2.2.1. α-Glucosidase

α-Glucosidase (otherwise known as α-1,4-glucosidase; IUMB enzyme nomenclature number EC 3.2.1.20 [45]), from baker’s yeast (*Saccharomyces cerevisiae*) Type I, belongs to the Glycoside Hydrolase 13 (GH_13) family [46], whose specificity is directed mainly towards the exohydrolysis of (1→4)-α-glucosidic linkages [47]. Under the conditions used (T = 37 °C; pH 6.8), α-glucosidase catalyzes the cleavage of the *p*-nitrophenyl-α-D-glucopyranoside (*p*NPG) substrate, resulting in the formation of α-D-glucose and *p*-nitrophenol (*p*Np). The amount of yellow color *p*Np liberated during the reaction was monitored by absorbance measurements at λ = 405 nm [48]

**Figure 2:**
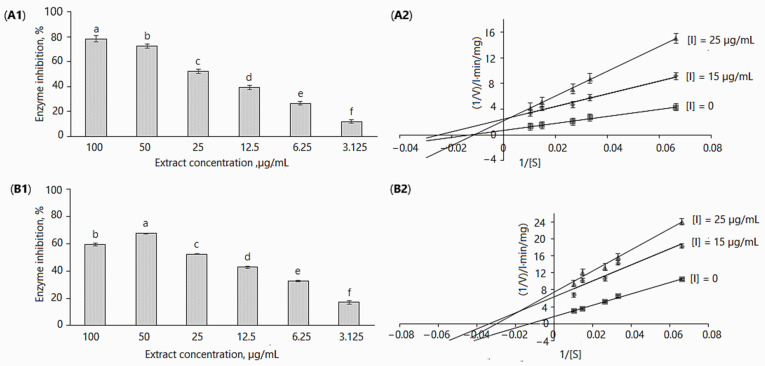
Inhibition of α-glucosidase by *D. caucasica* 70% ethanol leaf extracts at different concentrations (**A1**,**B1**) and Lineweaver–Burk plot using 0.05–0.33 mM of *p*NPG (**A2**,**B2**): (**A1**,**A2**) without adding 0.1 M Na_2_CO_3_; (**B1**,**B2**) with adding 0.1 M Na_2_CO_3_. The values are the means ± standard deviation SD (*n* = 3). Different letters in (**A1**,**B1**) indicate that the values are significantly different, according to Fisher’s LSD test (*p* < 0.05) after one-way ANOVA.

All in vitro experiments were performed at extract concentrations of 3.125, 6.25, 12.5, 25, 50 and 100 µg/mL. The extracts inhibited α-glucosidase activity in a dose-dependent manner; in the assays without adding 0.1 M Na_2_CO_3_ (Figure 2A1 and Table 2), enzyme activity as compared to control (without inhibitor) was reduced from 11.94 ± 1.22% to 78.49 ± 2.39% by increasing the concentration from 3.125 to 100 µg/mL. In case of the addition of 0.1 M Na_2_CO_3_, enzyme inhibition for the maximal and minimal applied concentrations was 59.30 ± 1.1% and 16.93 ± 1.43% (Figure 2A2 and Table 2). It may be observed that the differences in enzymatic activity with/without using 0.1 M Na_2_CO_3_ were not remarkable; however, they were statistically significant, except for the 25 µg/mL concentration (Table 2). Obviously, these differences may be related to the concentration of biologically active and other compounds present in the extracts.

**Figure 3:**
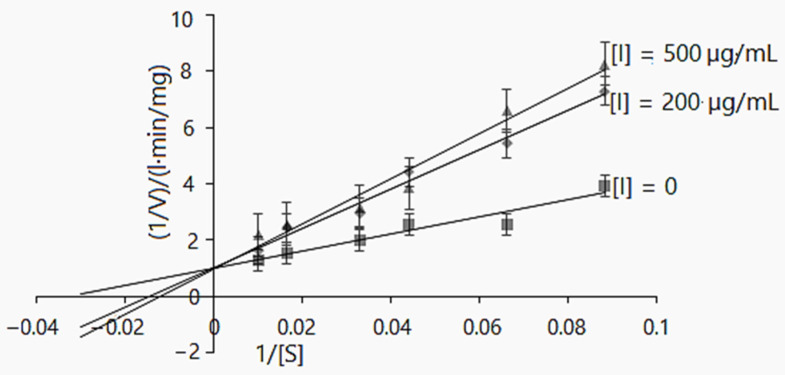
Lineweaver–Burk inhibition plot of α-glucosidase enzyme activity using *D. caucasica* tuber extract and different concentrations of *p*NPG (0, 0.004, 0.005, 0.008, 0.01, 0.02 and 0.33 mM). The signs and symbols on the graphic refers: squares: none inhibitor, rhombuses: 200 µg/mL of tubers extract concentration; triangles: 500 µg/mL of tubers extract concentration, [I]: inhibitor/extract; [S]: substrate. The values are the means ± SD (*n* = 3).

The IC_50_ values of *D. caucasica* leaf extracts with/without 0.1 M Na_2_CO_3_ at the tested concentrations were 41.99 µg/mL and 47.95 µg/mL, respectively. For comparison, acarbose at 200 µg/mL concentration reduced α-glucosidase activity by 71.17%. Most likely, the high inhibitory activity of *D. caucasica* leaf extracts may be attributed to the chlorogenic acids, quercitrin and isoquercitrin, although the contribution of other non-identified compounds cannot be discounted. In contrast, the tuber extracts of *D. caucasica* displayed rather weak inhibitory activity against α-glucosidase; for instance, at 500 µg/mL, enzyme activity was reduced by 40.79 ± 2.71% (Figure 3).

#### 2.2.2. α-Glucosidase Inhibition Kinetics

A good deal of information about enzymatic reactions can be obtained by observing the changes in the reaction caused by the presence of inhibitors. Based on the inhibitory results of the yam extracts used in this study, the mode of inhibition of α-glucosidase activities was investigated via kinetic studies in the absence and the presence of the inhibitor. The Lineweaver–Burk inhibition plots [49] of 1/V versus 1/[*p*NPG] gave the following equations: (1) without inhibitor: y = 55.052x + 0.7009, r^2^ = 0.9935; 15 µg/mL leaf extract: y = 99.316x + 2.482, r^2^ = 0.9852; 25 µg/mL leaf extract: y = 193.4x + 2.2253, r^2^ = 0.9989; (2) without inhibitor: y = 30.698x + 0.9772, r^2^ = 0.9292; 20 µg/mL tuber extract: y = 69.91x + 1.0085, r^2^ = 0.9833; 50 µg/mL tuber extract: y = 80.37x + 0.967, r^2^ = 0.966 (Figure 2A2). Similarly, a double reciprocal plot (Lineweaver–Burk plot) was constructed when the enzymatic reaction was stopped with 0.1M Na_2_CO_2_ (Figure 2B2). The following mathematical equations were obtained: (1) without inhibitor: y = 134.64x + 1.7083, r^2^ = 0.9943; 15 µg/mL of leaf extract: y = 188.35x + 6.3407, r^2^ = 0.9072; 25 µg/mL leaf extract: y = 249.6x + 7.4252, r^2^ = 0.9862.

*D. caucasica* leaf extracts displayed a mixed-type non-competitive mode of inhibition of α-glucosidase (Figure 2A2,B2). By virtue of the Lineweaver–Burk plot in the enzymatic reaction system I (Table 3), the V*_max_*_(*app*)_ decreased from 0.157 ± 0.03 to 0.134 ± 0.004 mg/L·min after increasing the inhibitor concentration from 15 to 25 µg/mL; K*_m_*_(*app*)_ values were 29.76 ± 1.54 and 33.61 ± 0.75 mg/L, respectively. In contrast, in the enzymatic reaction system II (Table 3), the increase in extract concentration resulted in an increase in the V*_max_*_(*app*)_ value, from 0.402 ± 0.041 to 0.449 ± 0.026 mg/L·min. The K*_m_*_(*app*)_ value in this case was more than two-fold higher (86.91 mg/L) at 25 µg/mL than at 15 µg/mL (39.984 mg/L). It was established that an increased K*_m_* and unchanged V*_max_* show competitive inhibition, while the decreased V*_max_* and increased/decreased K*_m_* values (Table 3) indicate the mixed-type inhibition model. Despite significant differences in kinetic constant (V*_max_*_(*app*),_ K*_m_*_(*app*)_) values among enzymatic reaction systems, noticeable is the hallmark of non-competitive inhibition. The double reciprocal plot showed a group of straight lines with different slopes, which intersect at the third quadrant (Figure 2B2)/second quadrant (Figure 2A2), suggesting that the extracts act as mixed-type inhibitors [50].

The catalytic efficiency (CE) of α-glucosidase in the different enzymatic reaction systems decreased as follows: in enzymatic reaction I, from 0.007 ([I] = 0) to 0.005 ([I] = 15 µg/mL) and 0.004 ([I] = 25 µg/mL); in enzymatic reaction II, from 0.018 ([I] = 0) to 0.01 ([I] = 15 µg/mL) and 0.005 ([I] = 25 µg/mL) (Table 3). The decreased rate of catalysis confirms the interaction of the inhibitor with α-glucosidase, which reduces product formation.

The kinetic reaction model was also elaborated for *D. caucasica* tuber extract. At the concentrations of 200 and 500 µg/mL, it competitively inhibited α-glucosidase: the K*_m(app_*_)_ values increased from 69.34 ± 0.31 to 83.33 mg/L, whereas V*_max(app)_* 0.99 ± 0.22 and 1.034 ± 0.38 mg/L·min were not significantly different (Table 3). Further, the Lineweaver–Burk plot provides information about 1/V*_max_* and −1/K*_m_* for the α-glucosidase kinetics. As may be observed (Figure 3), the increased K*_m_* value and insignificant changes in the V*_max_* value in the case of increasing inhibitor concentrations suggests the mechanisms of a competitive mode of inhibition [50,51]

#### 2.2.3. α-Amylase

**Figure 4:**
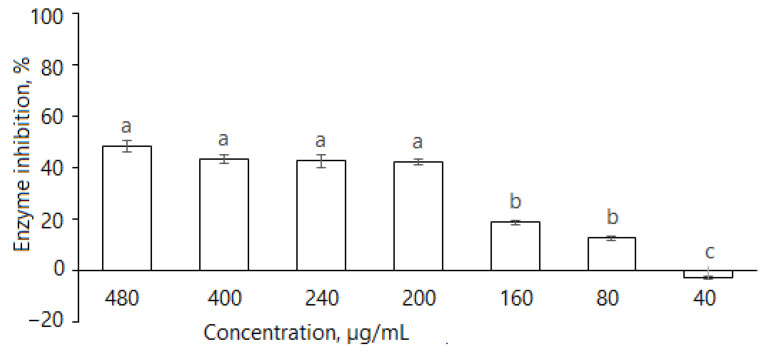
Inhibition of α-amylase by *D. caucasica* 70% ethanol leaf extracts at different concentrations (enzyme activity in the absence of extract was considered as 100%). The values are the means ± standard error (SE) (*n* = 3); different letters indicate that the values are significantly different, according to Fisher’s LSD test (*p* < 0.05) after one-way ANOVA.

As the tuber extract possessed remarkably weaker α-glucosidase inhibitory effects, further studies were focused on the leaf extract. It is evident that *D. caucasica* leaf extracts inhibited the α-amylase enzyme (Figure 4); at 480, 400, 240, 200, 160 and 80 μg/mL concentrations, it reduced α-amylase activity by 48.6 ± 2.2%, 43.4 ± 1.7%, 42.6 ± 2.3%, 42.2 ± 1.3%, 19.0 ± 0.9% and 12.8 ± 0.9%, respectively. The inhibitory effect of the plant was compared with the standard drug, acarbose, which reduced enzyme activity by approximately 93.8% at the concentration of 50 µg/mL. The inhibition increased with extract concentration; however, there was no linear dependence. At the concentration of ≥200 μg/mL, the changes in percentage inhibition were not significant. Most likely, it depends on the substrate composition and the mode of inhibition, which will be demonstrated further in the studies of reaction kinetics with other two tested enzymes. From these data, we can conclude that *D. caucasica* leaf extract was a moderate inhibitor (40–50% inhibition at ≥200 μg/mL concentration) of α-amylase, with a remarkably lower effect than acarbose.

#### 2.2.4. Acetylcholinesterase (AChE) Inhibition Assay

AChE (IUMB enzyme nomenclature number EC 3.1.1.7), also known as acetylcholine hydrolase, choline esterase I, cholinesterase, acetylthiocholinesterase and acetyl-β-methylcholinesterase, belongs to the cholinesterase (ChEs) family, which is most widely known for hydrolyzing the neurotransmitter acetylcholine (ACh) to choline (Ch) and acetic acid in the synaptic cleft. The principle of this assay method is based on the production of tiocholine (SCh), which is formed from acetylthiocholine during enzymatic hydrolysis with Ellman’s reagent (DTNB) and produces yellow-colored chromophore 5-thio-2-nitrobenzoate. The rate of appearance of the yellow derivative is measured spectrophotometrically at 412 nm [52].

The samples for the assay were prepared from the lyophilized powder and tested for their ability to inhibit AChE catalysis in the concentration range of 25–100 µg/mL. The results are listed in Table 4.

It may be observed that the inhibition noticeably increased after increasing the concentration of the leaf extract from 25 µg/mL (inhibition = 27.55%) to 40 μg/mL (inhibition = 40.58%), while a further increase in the concentration to 80 µg/mL resulted in a significant increase in AChE inhibition that was, however, less remarkable. However, at 80 and 100 µg/mL extract concentrations, there were no significant (*p* > 0.05) differences in decreased enzyme activity. The inhibitory effect of *D. caucasica* solution on AChE was compared with the synthetic inhibitor Donepezil HCl. As expected, the positive control was a more potent AChE inhibitor than the extracts of *D. caucasica*: at a 16 µg/mL concentration, it reduced enzyme activity by 70.62 ± 2% and V_max_ to 0.0593 µM/min/mg protein.

#### 2.2.5. Acetylcholinesterase Inhibition Kinetics

**Figure 5:**
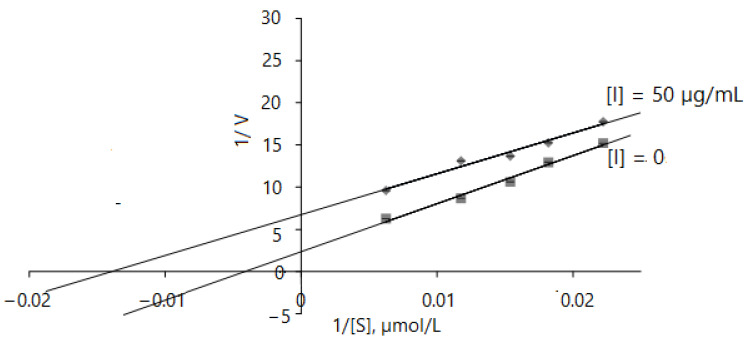
Mathematical modeling of Lineweaver–Burk inhibition plot on enzyme activity using different concentrations of ATChI (0, 45, 55, 65, 85 and 160 µM). The signs and symbols on the graphic refers: squares: none inhibitor, rhombuses: 50 µg/mL of leaves extract concentration; [I]: inhibitor/extract; [S]: substrate. Values represent mean ± standard deviation of triplicate samples.

The results obtained indicate that the AChE inhibition mechanism of *D. caucasica* leaf extracts may be rather complex; therefore, enzyme inhibition kinetic studies were performed by monitoring enzyme activity at varying concentrations of acethylthiocholine iodide in the range of 45–160 µmol/L. The Lineweaver–Burk inhibition plots of 1/V versus 1/[ATChI] gave the following equations: without inhibitor: y = 569.49 + 2.3524, r^2^ = 0.9874; with 50 µg/mL of leaf extract: y = 483.75 + 6.7466, r^2^ = 0.9755. *D. caucasica* leaf extracts displayed an uncompetitive mode of inhibition with respect to AChE (Figure 5). By virtue of the Lineweaver–Burk plot in the enzymatic reaction system with pure enzyme, the V_max,_ K_m_ values were 0.425 ± 0.11 µM/mg protein/min and 242.08 ± 15.76 µmol/L, respectively; meanwhile, in the case of using extracts at the concentration of 50 µg/mL, the V _max(app),_ K_m(app)_ values were remarkably lower, 0.148 ± 0.015 µM/min/mg protein and 71.56 ± 6.55 µmol/L, respectively. This profile of inhibition indicates that the inhibitor only binds to the enzyme–substrate complex [51].

#### 2.2.6. Angiotensin-Converting Enzyme (ACE)

**Figure 6:**
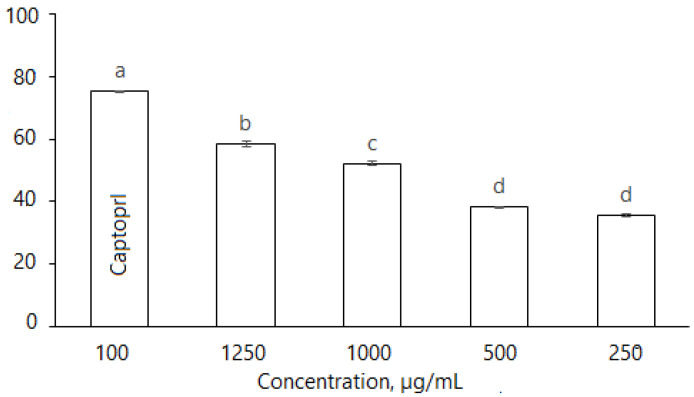
Inhibition of ACE by *D. caucasica* 70% ethanol leaf extracts at different concentrations (Captopril—positive control; negative control—no inhibition). Enzyme inhibition was expressed as percentage versus control (100%) (Control = 0 inhibition). The values are the means ± standard error (SE) (*n* = 3). The values with different letters are significantly different, according to Fisher’s LSD test (*p* < 0.05) after one-way ANOVA.

*D. caucasica* leaf extracts also inhibited ACE in a dose dependent manner (Figure 6): at the concentrations of 250, 500, 1000 and 1250 μg/mL, the enzyme inhibition was 35.46 ± 0.76%, 38.45 ± 0.52%, 51.12 ± 0.81% and 58.34 ± 1.01%. The inhibitory effect of plant extracts was compared with the synthetic antihypertension drug Captopril; at 100 µg/mL, the inhibition level was 74.3 ± 0.57%, while the effective concentration IC_50_ (ACE inhibition 50%) for the botanical extract was 829.7 µg/mL. Nevertheless, *D. caucasica* leaf extract may be considered a promising ingredient for the soft control of low/moderate hypertension.

## 3. Discussion

The search for and evaluation of biologically active nutrients in under-investigated plant materials is an important task, which may serve as a good platform in developing new effective ingredients for nutraceuticals and functional foods. In general, studies on the enzyme inhibitory activities of *Dioscorea* preparations are rather scarce. For instance, the extracts of *D. alata* and D*. bulbifera* tubers were tested as α-glucosidase and α-amylase inhibitors [20,53]. Our study substantially expands the knowledge on phytochemicals in *D. caucasica* leaves and tubers and their effects on physiologically important enzymes, namely α-amylase, α-glucosidase, angiotensin-converting enzyme and acetylcholinesterase. It is evident that the polyphenolic-rich extract of leaves is a remarkably stronger inhibitor of all tested enzymes than that of the tubers. Sterols (campesterol, sitosterol, stigmasterol), triterpene acids (oleanolic and ursolic acid), pentacyclic triterpenoids and their esters (α-amyrin, β-amyrin, taraxasterol, taraxerol) were reported in previously published articles on *D. caucasica* leaf constituents [25], while saponins such as parvifloside, protodeltonin, protodioscin, deltonin, dioscin [54] and diosgenin [54,55] were the dominant compounds in plant tubers. Some studies reported the hypoglycemic and α-glucosidase/α-amylase inhibitory activities of phytosterols [56] present in seed oils and triterpenoid saponins from botanicals [57]; however, it is highly unlikely that liposoluble (hydrophobic) leaf phytochemicals play a significant role as enzyme inhibitors. Therefore, the stronger enzyme inhibition by leaf extracts may be related to the presence of the main polyphenolic antioxidants, 3-*O*-glycoside flavonoid and hydroxycinnamic acid derivatives (Table 1).

Inhibiting the activity of amylolytic enzymes is important in controlling the postprandial glycemic index, which may help in managing type-2 diabetes mellitus. Chlorogenic and neochlorogenic acids and quercetin derivatives are well-known inhibitors of α-glucosidase and α-amylase [58,59]. For instance, the inhibition of α-glucosidase very strongly correlated with chlorogenic acid present in the young inflorescence tissues of selected Rosaceae plants [60]. However, the inhibitory activity depends on the structural peculiarities of the phenolic compounds. Recently, Zhang et al. [61] reported that the fraction of bound polyphenolics of red quinoa composed mainly of ferulic acid more strongly inhibited α-glucosidase than the fraction of free polyphenolics consisting mainly of hydroxybenzoic acid and its derivatives; the former fraction inhibited α-glucosidase in an uncompetitive mode, while the latter one in a non-competitive mode. This was explained by the peculiarities of the interactions between ferulic acid and enzyme amino acid sites. Flavonols such as quercetin were also reported as potent inhibitors of α-amylase and α-glucosidase in an activity-guided study of chokeberry, pomegranate and red grape extracts [62]. Based on the strong inhibition of glucose release from complex carbohydrates, herbal infusions containing phenolic acids were suggested as natural preparations for preventing type II diabetes [6]. The hypoglycemic effect of *di*-caffeoylquinic acid derivatives may be also explained by the modulation of α-glucosidase, while chlorogenic acid is a potent inhibitor of glucose 6-phosphate translocase [63].

Quite interesting behavior was observed by evaluating extract-induced α-glucosidase inhibition (Table 2) and reaction kinetics (Figure 2 and Table 3). It may be noted that the main findings were valid regardless of some modification of the method described in the Sigma-Aldrich protocol [48]. Our data suggest that using 0.1 M Na_2_CO_3_ solution may significantly change the manner of extract-mediated α-glucosidase inhibition. Significantly negative differences in the activity shift were observed at concentrations of up to 25 µg/mL, while, at higher concentrations, the inhibition in the reaction without 0.1 M Na_2_CO_3_ was remarkably higher. To the best of our knowledge, the peculiarities of botanical extract-induced α-glucosidase inhibition using this approach have not been reported previously. Most likely, the significant differences in inhibition between the reaction systems, which were particularly clearly pronounced in the case of increasing extract concentrations, were due to changes in the ratios of the compounds with different effects on the enzyme.

Lineweaver–Burk, Eadie–Hofstee and Hanes–Woolf plots are widely used for determining important parameters in enzyme kinetics, although they may result in some erroneous data [64]. Chon et al. [65] compared five estimation methods, including Lineweaver–Burk and Eadie–Hofstee plots, for simulation data using additive error and combined error models for the parameters of the Michaelis–Menten equation and concluded that the estimation of V*_max_* and K*_m_* by nonlinear methods provided the most accurate results, while in case of linear methods, the Eadie–Hofstee plot exhibited some advantages over the Lineweaver–Burk plot, although, for the simulation data incorporating additive error, estimated V*_max_* values were similar. In addition, it was noted that the double reciprocal Lineweaver–Burk plot overestimates rate measurements recorded at low substrate concentrations, where the experimental error is liable to be greatest [66], while the Eadie–Hofstee plot is less biased at low [S]; however, it can result in significant errors since both coordinates contain V [64]. Marasović et al. (2017) [67] suggested that the Hanes–Woolf plot is the most accurate of the three; however, its major drawback is that both ordinate and abscissa are dependent on the substrate concentration. Despite the perceived errors of the described methods, they are all presented in the scientific literature and are applied for determining kinetic parameters. We chose the most widely used Lineweaver–Burk method, which enabled us to demonstrate that the *D. caucasica* leaf and tuber extracts displayed mixed-type and competitive modes of inhibition of α-glucosidase, respectively.

Numerous plant origin bioactive compounds and food-derived peptides may inhibit ACE in a very wide range of IC_50_. Regarding yams, *D. opposita* autolysate and enzymatic hydrolysates of tuber mucilage were tested as ACE inhibitors [68], while the inhibitory properties of leaf extracts have not been reported. *D. causacica* leaf extract inhibited ACE in a dose-dependent manner (Figure 6). Polyphenolic compounds in this case may also play the most important role. For instance, wine lees of Cabernet grape variety, which contained significantly higher amounts of catechin flavanols than other evaluated varieties, effectively inhibited ACE and demonstrated similar potency to the drug Captopril in hypertensive rats [69]. In vivo studies with mice showed that a dietary supplement consisting of selected botanicals, chlorogenic acid and inulin reduced the risk factors of cardiovascular diseases by lowering hepatic angiotensinogen and angiotensin-II levels, among other factors [70]. The hypotensive effects of the phenolic-rich fraction of red wine were also demonstrated by an in vivo study with spontaneously hypertensive rats [5].

The AChE enzyme is involved in numerous noncholinergic physiological functions, such as promoting cell development and differentiation, participating in apoptosis and relating to pathogenic processes including Alzheimer’s disease and tumorigenesis [71]. Various compounds, e.g., galanhtamine, huperizine A, *N*-demethyl-puqietinone, yibeinoside A, quercetin, etc., which are abundant in some plant species, were reported as AChE inhibitors [4,72]. The anti-cholinesterase ability of *Phyllanthus emblica* fruit extract was also related to a high amount of flavonols and phenolic acids [73], which were abundant in the *D. caucasica* leaf extract evaluated in our study. The diethyl ether and ethyl acetate extracts of *D. communis* tubers tested in previous studies did not show an AChE inhibitory effect; however, some individual *Dioscorea* constituents (2,3,4-trimethoxy-7,8-methylenedioxyphenanthrene, 2,4-dimethoxy-7,8-methylenedioxy-3-phenanthrenol, 2,4,8-trimethoxy-3,7-phenanthrene-diol) inhibited the enzyme [3]. Phenanthrene derivates we not detected in ethanol extracts, but the possibility cannot be ruled out that some other nonidentified compound may have inhibited this enzyme. For example, Tenfen et al. (2019) [74] reported that the strong inhibition of *Eugenia* genus leaf extracts against AChE was associated with the presence of isoquercitrin, quercetin, catechin, epicatechin, procatecuic acid and myricitrin content. Thus, the extract from the leaves of *D. caucasica* inhibits the AChE enzyme in a dose-dependent manner, as with other, previously tested enzymes.

## 4. Materials and Methods

### 4.1. Reagents

α-Glucosidase (EC 3.2.1.20) from *Saccharomyces cerevisiae* Type I; lyophilized powder, ≥10 units/mg protein (G5003—100 UN) [75]; α-amylase (EC 3.2.1.1) from porcine pancreas (Sigma A6255, 1151 U/mg of protein) [76], lung acetone powder from rabbit (L-0756) [77], *N*-[3-(2-furyl)acryloyl]-L-phenylalanine-glycyl-glycine (FAPGG), *p*-nitrophenyl α-D-glucopyranoside (*p*NPG) (N1377-1G), 5,5′-dithiobis-(2-nitrobenzoic acid) (DTNB, ≥99%), acetylthiolcholine iodide (ATChI); and starch were obtained from Sigma-Aldrich (St. Louis, MO, USA). Acetylcholinesterase (EC 3.1.17) from *Electrophorus electricus*, Type VI-S; lyophilized powder, 200–1000 units mg/protein (C3389) [78], was from Sigma-Aldrich Chemie (Taufkirchen, Germany); 3.5-dinitrosalicilyc acid (DNS) from Acros Organics (Geel, Belgium), 4-nitrophenol (C_6_H_5_NO_3_) from Alfa Aesar (Kandel, Germany). Potassium phosphate dibasic (K_2_HPO_4_), potassium phosphate monobasic (KH_2_PO_4_), sodium phosphate monobasic (NaH_2_PO_4_), sodium phosphate dibasic (Na_2_HPO_4_), tris(hydroxymethyl) aminomethane hydrochloride, boric acid (H_3_BO_3_), anhydrous sodium carbonate (Na_2_CO_3_), sodium chloride (NaCl) and sodium hydrocarbonate (NaHCO_3_) were purchased from Eurochemicals (Vilnius, Lithuania); 1M hydrochloric acid (HCl) from Merck KGaA (Darmstadt, Germany). Agricultural origin ethanol (96.6 %) was from Stumbras (Kaunas, Lithuania). Captopril, 1-[(2S)-3-mercapto-2-methyl propionyl] (Salutas Pharma GmbH, Barleben, Germany) and donepezil hydrochloridum (Accord Healthcare Limited, London, UK) were purchased from the local pharmacy. Acarbose (C_25_H_43_NO_18_) was from Merck KGaA (Darmstadt, Germany); reference compounds, neochlorogenic acid, chlorogenic acid, rutin and quercitrin were from Sigma Chemical Co. (St. Louis, MO, USA). Other chemicals and reagents used in the current study were of analytical grade and obtained from Sigma-Aldrich (St. Louis, MO, USA), unless other sources are indicated. Ultrapure water was produced using a Simplicity 185 system (Millipore, MA, USA). All buffers were made using ultrapure water and pH measurements were performed at room temperature using an Agilent 3200P pH Meter (Agilent Technologies, Inc., Shanghai, China).

### 4.2. Preparation of D. caucasica Extracts

*Dioscorea caucasica* Lipsky leaves and tubers were collected from the Kaunas Botanical Garden of Vytautas Magnus University in Lithuania (55°52′14′′ N 23°54′37′′ E) [79] in June 2019. The collected materials were air-dried at room temperature and milled in a centrifugal mill, Retsch ZM200 (Haan, Germany), using a sieve with 0.5 mm diameter holes. Twenty grams of powdered sample were extracted with 200 mL 70% (*v*/*v*) ethanol in a rotary shaker (200 rpm) during 14 h at room temperature. The extract obtained was filtered through Whatman filter paper no. 1 (Whatman^®^ International Ltd., Maidstone, UK) to obtain the first extract. This procedure was repeated on the residue using 100 mL 70% ethanol for 1 h to obtain the second filtrate. Both filtrates were combined and the solvent was removed in a rotary vacuum evaporator, the Büchi Rotavapor R-210 (Büchi Labortechnik, Flawil, Switzerland), at 40 °C. The residual water was evaporated by freeze-drying. The yield of dry extract powder was 17.86% and 15.65% from leaves and tubers, respectively. Freeze-dried extracts (100 ± 0.1 mg) were dissolved in 10 mL of 70% (*v*/*v*) ethanol in a volumetric flask to prepare 10 mg/mL extract stock solutions, which were stored at 4 °C until further use.

### 4.3. Identification and Quantitative Analysis of the Main Phytochemicals

#### 4.3.1. Identification of Phytochemicals by UPLC-QTOF-MS

The extracts were separated using a Waters Acquity UPLC system [80,81] consisting of a binary solvent manager, autosampler, column heater and a photodiode-array detector (PDA) (Waters Corporation, Milford, MA, USA). A Waters Acquity BEH C18 (100 × 2.1 mm; 1.7 μm) [82] column was used for compound separation. Eluent A was 0.4% (*v*/*v*) formic acid solution in ultrapure water, and B was acetonitrile. The gradient was formed as follows: initially, the separation was started with 100% A; then, in 9 min, B was increased to 100%, and was held at 100% for 1 min. After this, the column was returned to initial conditions in 1 min and then was allowed to equilibrate for 1 min. The column was equilibrated for 2 min before each run. Flow rate was 0.4 mL/min, injection volume 1 µL and column temperature 40 °C.

The eluted compounds were analyzed on a MAXIS 4G QTOF mass spectrometer (Bruker Daltonik GmbH, Bremen, Germany) equipped with an electrospray ionization (ESI) source. The spectrometer was operated in negative ionization mode, and capillary voltage was maintained at 4000 V. Nitrogen (N_2_) was used as a nebulizing and drying gas at 2.5 bar pressure and 10 L/min flow rate. Drying gas temperature was maintained at 200 °C. MS were recorded in the range from 80 to 1200 *m*/*z*; spectra recording rate was 3 Hz. The compounds profiles were identified by comparing MS results with previously reported data.

#### 4.3.2. Quantification of the Main Phytochemicals by HPLC

The extracts were prepared by dissolving 200 ± 0.1 mg of freeze-dried powdered leaves/tubers in 10 mL of methanol in an ultrasonic bath for 30 min at 20 ± 0.2 °C. Subsequently, the aliquots of 10 µL samples were injected into the column of a Waters Alliance 2695 HPLC, and the oven temperature was kept at 25 °C. The mobile phase A (0.1% trifluoroacetic acid (*v*/*v*) in ultrapure water) and mobile phase B (acetonitrile) were operated with a fast eluting gradient as follows: 0–8 min, 95% A, 5% B; 8–30 min, 90–80% A, 10–20% B; 20–30 min, 80–60% A, 20–40% B; 30–40 min, 60–40% A, 40–60% B; 40–45 min, 20–40% B; 30–40 min, 60–40% A, 40–60% B; 40–45 min, 40–30% A, 60–70% B; and 45–50 min, 30–90% A, 70–10% B. Chromatographic separation was performed in an Atlantis C18 column (250 mm × 4.6 mm i.d., 5 µm). Detector was set at 320 nm for phenolic acids (neochlorogenic and chlorogenic), 360 nm for flavonoids (rutin, isoquercitrin). Neochlorogenic acid, chlorogenic acid, rutin and isoquercitrin were eluted at 9.84, 12.28, 23.43 and 25.55 min, respectively (Figure 1). Quantitative determination was performed by using calibration curves, which were produced using reference compounds.

### 4.4. Preparation of Solutions for Enzyme Inhibition Assays

#### 4.4.1. Rabbit Lung and Captopril Solutions for Angiotensin-Converting Enzyme (ACE) Inhibition Assays

Rabbit lung acetone powder [76] was prepared as described by Vermerissen et al. (2002) [83], with slight modification. Briefly, 100 ± 0.1 mg of powder was dissolved in 100 mL of cold borate buffer (80 mmol/L, pH 8.3 ± 0.05) and kept at 5 °C temperature overnight. Insoluble matter was centrifuged in a centrifuge, the MPW 260RH (Med. Instruments, Warsaw, Poland), at 6000 rpm for 30 min at 4 °C temperature. After centrifugation, the clear wine-red supernatant was carefully transferred into a clean test tube and used for experiments.

Captopril solution was prepared as described by Donath-Nagy et al. (2011) [84], with slight modifications. Two tablets containing 25 mg of Captopril were ground in a mortar and extracted with approximately 15 mL water in a 25 mL volumetric flask in the ultrasonic bath (Bandelin Sanorex, Berlin, Germany) for 10 min, and then brought to volume with water and filtered through Whatman filter paper. Solution of Captopril (1 mg/mL) was used as a positive control in the ACE inhibition assay.

#### 4.4.2. Donepezil HCl Solution for Acetylcholinesterase (AChE) Inhibitory Activity

Donepezil hydrochloride solution was prepared from two tablets containing 5 mg of Donepezil Accord drug. The tablets were crushed and 100 ± 0.1 mg of fine powder was dissolved in 100 mL of ultrapure water in a 100 mL volumetric flask to obtain a 1 mg/mL Donepezil HCl stock solution. Working solutions of Donepezil HCl were obtained by the appropriate dilution of the stock solution and used as positive controls in AChE inhibition assays.

#### 4.4.3. Acarbose Solution for α-Amylase and α-Glucosidase Inhibition Assays

Aqueous solution of acarbose was prepared by dissolving 100 ± 0.1 mg acarbose in a 100 mL volumetric flask to obtain a 1 mg/mL acarbose stock solution. Working solutions of acarbose were obtained by appropriate dilution of the stock solution and used as positive controls in α-amylase/α-glucosidase inhibition assays.

### 4.5. Determination of Enzyme Inhibition Activities

#### 4.5.1. α-Glucosidase

The α-glucosidase inhibition was evaluated by the chromogenic method using *p*NPG as a substrate [48]. The α-glucosidase activity measurements were conducted at 37 ± 0.2 °C using a 1 mL mixture composed of 0.33 mM *p*NPG, dissolved in potassium phosphate buffer (pH 6.8) and different concentrations of inhibitors, namely 0, 3.125, 6.25, 12.5, 25, 50 and 100 µg/mL. The assay was initiated by the addition of 5 µL solution of enzyme. α-Glucosidase and product formation (*p*NPG + α-glucosidase → α-D-glucose + *p*Np was monitored after 30 min at 405 nm using the Spectronic Genesys 8 spectrophotometer (Thermo Spectronic, Rochester, NY, USA). The data were recorded in two ways: with the stop solution 0.1 M Na_2_CO_3_ and without it. The control solution was prepared using the same buffer without inhibitor. The percentage of inhibition of α-glucosidase was calculated similarly to α-amylase.

For kinetic studies, α-glucosidase activity was measured spectrophotometrically by following the absorbance at 405 nm in assay mixture containing various concentrations of substrate: 0.1mM, 0.125 mM, 0.23 mM and 0.33 mM *p*NPG in 0.1 M K_2_HPO_4_/ KH_2_PO_4_ phosphate buffer, pH 6.8 ± 0.05, fixed amount of α-glucosidase (5 µL) solution and the absence or presence of the leaf and tuber extracts at 15 µg/mL, 25 µg/mL and 200 and 500 µg/mL concentrations, respectively.

#### 4.5.2. α-Amylase

In vitro α-amylase inhibition was assayed by the method by described in the Sigma-Aldrich protocol [85], with slight modifications. α-Amylase [76] was diluted (1:1000) in 0.02 mM sodium phosphate buffer (pH 6.9) containing 6.7 mM sodium chloride to produce stock solution. The reaction mixture containing 400 µL of α-amylase (0.5 IU/mL) and 400 μL of varying concentrations of extracts (40–480 µg/mL) was incubated in a test tube at 25 °C ± 0.2 °C for 30 min, followed by the addition of 400 µL of 1% (*w*/*v*) potato starch solution (0.02 mM sodium phosphate buffer, pH 6.9 ± 0.5), as a substrate, and further incubation for 3 min. The reaction was terminated by the addition of 800 µL of 3.5-dinitrosalicylic acid (DNSA) and heating in a water bath for 10 min at 85 ± 0.2 °C. Afterwards, the mixture was removed from the water bath, cooled under tap water and diluted with 3 mL of distilled water. The absorbance (A) was measured at 540 nm and the inhibition activity was calculated by the following equation: % Inhibition = (A_control_ − A_sample_)/A_control_ × 100. The blank sample was prepared without enzyme; control samples were those without extracts (100% enzyme activity). All tests were performed in triplicate. Acarbose was used as a positive control.

#### 4.5.3. Acetylcholinesterase (AChE)

The AChE inhibition was estimated in vitro by Ellman’s method [52] using ATChI as a substrate. The spectrophotometric method for the estimation of AChE activity is based on the determination of yellow-colored chromophore (5-thio-2-nitrobenzoate; C_7_H_5_NO_4_S; λ = 412 nm) during enzymatic hydrolysis with DTNB reagent (5,5′-dithiobis-(2-nitrobenzoic acid), which reacts with the sulfhydryl groups of the protein. Briefly, AChE activity measurements were carried out at a constant temperature of 20 ± 0.5 °C using a 1 mL mixture composed of 50 mmol/L Tris/HCl (pH 8.0 ± 0.05) buffer, different concentrations (25–100 µg/mL) of inhibitor, 0.05 mmol/L DTNB and AChE at 0.55U. After pre-incubation for 10 min, the reaction was initiated by the addition of 2.5 mM ATChI. The formation of a DTNB-tios complex (C_7_H_5_NO_4_S) was measured at 412 nm by using a GENESYS 50 UV/Vis spectrophotometer (GENESYS Instruments Limited, Cambridge, UK). Donepezil, a standard AChE inhibitor, was used as a positive control and Tris/HCl (pH 8.0 ± 0.05) was used as a negative control. Enzyme-specific activity was calculated using nitrobenzoate (TNB) molar extinction coefficient ε =14150 M^−1^ cm^−1^ [86] and expressed as µmol thiocholine formed/min/mg protein. The enzyme percentage inhibition was calculated by comparing the enzymatic activity with/without inhibitor using the following equation: % I = (A_control_ − A_sample_) /A_control_ × 100 %, where A_contol_ is AChE activity for the negative control, and A_sample_ is the presence of the plant extract or Donepezil.

For kinetic studies, AChE activity was measured spectrophotometrically by following the absorbance at 412 nm in assay mixture containing various concentrations of substrate, 45, 50, 65, 85 and 160 µM ATChI, in 50 mmol/L Tris/HCl, pH 8.0 ± 0.05, fixed amount of AChE (10 µL) solution and the absence or presence of the leaf extracts at 50 µg/mL concentration.

#### 4.5.4. Angiotensin-Converting Enzyme (ACE)

ACE inhibition was estimated in vitro by measuring the release of *N*-[3-(2-furyl)acryloyl]-L-phenylalanine (FAP) and glycine-glycine (Gly-Gly) from the substrate FAPGG (C_20_H_21_N_3_O_6_) according to the simple, rapid and sensitive method of Vermerissen et al. (2002) [83]. Ethanol solutions of plant extract at concentrations of 250, 500, 1000 and 1250 µg/mL were used for the ACE inhibition assay. Three hundred µL of rabbit lung ACE (rabbit lung acetone powder reconstituted in 80 mM sodium borate buffer containing 300 mmol/L sodium chloride, pH 8.3 ± 0.05) and 200 μL of inhibitor solution were added into a test tube and pre-incubated for 3–5 min at room temperature. After pre-incubation, the reaction was started (t = 0 min) by adding 800 μL ACE-specific substrate solution comprising 0.8 mmol/L FAPGG dissolved in a borate buffer, to the final volume of 1300 μL. The mixture was allowed to stand at room temperature for 1 min and absorbance was recorded at 340 nm using the Biochrom Libra S4+ visible spectrophotometer (Biochrom Ltd., Cambridge, UK). Subsequently, the absorbance was monitored after 20 min at 37 ± 0.2 °C. Hydrolysis of FAPGG results in a decrease in absorbance at 340 nm. The rate of decrease in absorbance is directly proportional to ACE activity in the sample. The percentage inhibition of ACE activity by plant extracts was calculated with the following equation: Inhibition, % = [100 − (_Δ_A_340/min_ inhibitor/_Δ_A_340/min_ control × 100], where _Δ_A is the difference between the initial and final absorbance of the test sample during its incubation.

### 4.6. Mathematical Modeling of Enzyme Inhibition Kinetics

The application of kinetic modeling can provide important insights into how interacting components behave in biological systems. The changes in enzyme catalytic activity in a biochemical system are frequently modeled by choosing a mathematical model to evaluate the following fundamental parameters: Michaelis–Menten constant (K*_m_*), maximal velocity (V*_max_*) and kinetic constant (K*_i_*). In this study, kinetic data were estimated by virtue of plotting the data on a double reciprocal graph, called a Lineaweaver–Burk plot [49], to the following equation: 1/V = K*_m_*/V*_max_* × 1/[S] + 1/V_max_, where V is the reaction rate, K*_m_* is the Michaelis–Menten constant, V*_max_* is the maximum reaction rate (mg/L·min), [S] is the *p*NPG concentration (mg/L). The resultant plot is a straight line, with X- and Y-axis intercepts representing −1/K*_m_* and 1/V*_max_*, respectively, and the slope is K*_m_*/V*_max_*. The inhibition type of the inhibitors was determined by analyzing the Lineaweaver–Burk plots, which were competitive, non-competitive or uncompetitive, and the inhibition constant (K_i_) values for botanical extracts were estimated by fitting the equation K*_m_*_(*app*)_ = K*_m_* (1 + [I]/K_i_) for competitive inhibition and equation V*_max_*_(*app*)_ = V*_max_* (1 + [I]/K*_i_*) for non-competitive inhibition, where K*_m_*_(*app*)_ and K*_m_* are the concentrations of a substrate required to produce 50% of its maximum velocity (V*_max_*) in the presence and absence of an inhibitor, which were determined in parallel; [I] is the compound concentration; K*_i_* is the inhibition constant of the reactions. Table 5 summarizes the types of inhibition and their effects on these parameters.

### 4.7. Statistical Data Analysis

Data are expressed as mean ± standard error (SE) and/or standard deviation (SD). Reaction velocities and enzyme kinetics and IC_50_ values of the extracts were calculated using Microsoft Excel 2016. Statistical analysis was performed by Student’s *t*-test, paired-samples *t*-test and Fisher’s least significance difference (LSD) test (*p* < 0.05).

## 5. Conclusions

The first systematic phytochemical study of *D. caucasica* revealed that the plant leaves are rich in phenolic compounds, mainly caffeic acid esters and quercetin glycosides, while its tubers accumulate steroidal glycosides, as the major secondary metabolites. *D. caucasica* leaf extracts were remarkably stronger inhibitors of α-glucosidase, α-amylase, acetylcholinesterase and angiotensin-converting enzyme than the tuber extracts. Kinetic studies of enzyme inhibition suggest that the mode of inhibition, depending on the extract origin, may be mixed-type (leaves) and competitive (tubers). Based on the results obtained in this study and previously reported data, it may be sufficiently reasonably assumed that *D*. *caucasica* leaf polyphenolics play the most important role in the enzyme inhibition, while the tuber steroidal glycosides might be remarkably weaker enzyme inhibitors or their effects may be hampered by other, antagonistically competitive constituents. In general, the new data on *D. caucasica* phytochemicals and bioactivities may aid in the valorization of this plant for its wider use in the development of health beneficial ingredients for functional foods and nutraceuticals.

## Figures and Tables

**Table 1:** Identification data of the constituents detected in *D. caucasica* leaf and tuber extracts by UPLC/Q-TOF-MS in negative ion mode.

ID No.	t_R,_ min	*m*/*z* [M − H]^−^ Found	*m*/*z* [M − H]^−^ Calculated	Detected Ion(s)	Mass Error (ppm)	Compound	Leaves	Tubers	Reference
Organic Acids									
1	0.4	191.0561	191.0556	191[C_7_H_11_O_6_]^−^	−0.5	Quinic acid	+		
	0.7 ^#^	191.0562			−0.6			+	MS data
2	0.5	133.0142	133.0137	133[C_4_H_5_O_5_]^−^	−0.5	Malic acid	+		[26,30]
	0.8 ^#^	133.0142			−0.5			+	
3	0.5	173.0454	173.0450	173[C_7_H_9_O_5_]^−^	−0.4	Shikimic acid	+		[26]
4	0.8	191.0196	191.0191	191[C_6_H_7_O_7_]^−^	−0.4	Citric acid/isocitric acid	+		
	1.2 ^#^	191.0200			−0.8			+	MS data
5	1.8	255.0512	255.0505	255[C_11_H_11_O_7_]^−^	−0.7	Piscidic acid		+	[27]
**Hydroxycinnamates**									
6	1.5	707.1822	707.1823	707[C_32_H_35_O_18_]^−^ [2M − H]^−^	−0.1		+		
		353.0875	353.0872	353[C_16_H_17_O_9_]^−^	−0.3	3-Caffeoylquinic acid *			[31]
	1.6	707.1822	707.1823	707[C_32_H_35_O_18_]^−^ [2M − H]^−^	−0.1		+		
8		355.0664	355.0665	355[C_15_H_15_O_10_]^−^	−0.1	Caffeic acid *O*-glucuronide			
9		297.0612	297.0610	297[C_13_H_13_O_8_]^−^	−0.2	Caffeoylthreonic acid	+		[31]
10	1.7	707.1827	707.1823	707[C_32_H_35_O_18_]^−^ [2M − H]^−^	−0.4		+		
		353.0876	353.0872	353[C_16_H_17_O_9_]^−^	−0.4	5-Caffeoylquinic acid *			[26]
		191.0559	191.0556	191[C_7_H_11_O_6_]^−^	−0.5				
11	1.9	337.0930	337.0923	337[C_16_H_17_O_8_]^−^	−0.4	Coumaroylquinic acid	+		
12		353.0877	353.0872	353[C_16_H_17_O_9_]^−^	−0.5	4-Caffeoylquinic acid	+		[31,32]
		191.0564		191[C_7_H_11_O_6_]^−^	−0.7		+		
13	2.0	335.0771	335.0766	335[C_16_H_15_O_8_]^−^	−0.5	Caffeoylshikimic acid	+		[33]
14	2.1	367.1031	367.1029	367[C_17_H_19_O_9_]^−^	−0.2	Feruloylquinic acid	+		[31]
**Flavonoids**									
15	2.3	609.1463	609.1455	609[C_27_H_29_O_16_]^−^	−0.8	Quercetin-3-*O*-rutinoside (rutin) *	+		
16	2.4	463.0879	463.0876	463[C_21_H_19_O_12_]^−^	−0.3	Quercetin-3-*O*-glucoside (isoquercitrin) *	+		
17		505.0995	505.0982	505[C_23_H_21_O_13_]^−^	−1.3	Quercetin-*O*-acetylhexoside	+		[34]
18	2.5	549.0883	549.0880	549[C_24_H_21_O_15_]^−^	−0.3	Quercetin 3-*O*-(6′′-malonyl) glucoside	+		
19	2.6	447.0930	447.0927	447[C_21_H_19_O_11_]^−^	−0.3	Quercetin-3-*O*-rhamnoside (quercitrin)	+		[35]
20	2.7	533.0934	533.0931	533[C_24_H_21_O_14_]^−^	−0,27	Quercetin-3-*O*-malonyl(rhamnoside)	+		MS data
21		489.1036	489.1033	489[C_23_H_21_O_12_]^−^	−0.3	Quercetin-3-*O*-acetyl(rhamnoside)	+		
**Sugars**									
22	0.6	179.0561	179.0555	179[C_6_H_11_O_6_]^−^	−0.6	Hexose		**+**	
23	0.7	341.1089	341.1083	341[C_12_H_21_O_11_]^−^	−0.6	Sucrose		+	[26,36,37]
24	0.7	683.2252	683.2246	683[C_24_H_43_O_22_]^−^ [2M − H]^−^	−0.6	Unseparated sugars		+	[37]
25	0.7	1025.3414	1025.3408	1025[C_36_H_63_O_33_]^−^ [3M − H]^−^	−0.6	Unseparated sugars		+	[37]
**Fatty Acids and Conjugates**									
26	4.6	329.2335	329.2328	329[C_18_H_33_O_5_]^−^	−0.7	Trihydroxy octadecenoic acid		+	[38]
27	5.8	293.2120	293.2117	293[C_18_H_29_O_3_]^−^	−0.3	Hydroxy octadecatrienoic acid	+		[38]
28	6.8	358.2601	358.2593	358[C_19_H_36_NO_5_]^−^	−0.8	Hydroxy dodecanoylcarnitine		+	LMFA07070032 [39]
29	6.9 8.0	295.2280 295.2281	295.2273	295[C_18_H_31_O_3_]^−^	−0.7 −0.8	Hydroxy octadecadienoic acid (linolenic acid)		+	[38]
30	8.4	271.2279	271.2273	271[C_16_H_31_O_3_]^−^	−0.6	Hydroxy hexadecenoic acid		+	[38]
31	8.8	279.2330	279.2324	279[C_18_H_31_O_2_]^−^	−0.6	Octadecadienoic acid (Linoleic acid)		+	[38]
**Other Compounds**									
32	0.3	225.0617	225.0610	225[C_7_H_13_O_8_]^−^	−0.7	Glucoheptonic acid	+		[29]
33	0.9	290.0881	290.0875	290[C_11_H_16_NO_8_]^−^	−0.6	Neu5Ac2en	+		CID65309
34		128.0352	128.0347	128 [C_5_H_6_NO_3_]^−^	−0.5	Pyroglutamic acid	+		[28]
35	1.4	345.1188	345.1186	345[C_15_H_21_O_9_]^−^	−0.2	Aucubin	+		
36	3.3	1109.5383	1109.5379	1109[C_52_H_85_O_25_]^−^	−0.4	Steroidal glycoside		+	[40]
37	3.7	1093.5431	1093.5431	1093[C_52_H_85_O_24_]^−^	−0.0	Steroidal glycoside		+	[40,41]
38	4.3	1075.5327	1075.5325	1075[C_52_H_83_O_23_]^−^ [M − H-146]^−^	−0.2	Steroidal glycoside (ophiopogonin derivative?)		+	[40]
39	6.5	929.4754	929.4746	929[C_46_H_73_O_19_]^−^	−0.1	Steroidal glycoside		+	[40]
40	6.6	913.4798	913.4797	913[C_46_H_73_O_18_]^−^	−0.1	Steroidal glycoside		+	[40]
41	6.8	767.4228	767.4217	767[C_40_H_63_O_14_]^−^	−1.1	Steroidal saponin (pentandroside B?)		+	[42]
**Unidentified Compounds**									
42	6.1	559.3117	559.3118	559[C_28_H_47_O_11_]^−^	−0.1	Unidentified	+		MS data
43	6.6	483.2727	483.2719	483[C_25_H_35_N_6_O_4_]^−^	−0.8	Unidentified peptide	+		MS data
44	5.6	721.3652	721.3646	721[C_34_H_57_O_16_]^−^	−0.6	Unidentified galactolipid	+		MS data
45	6.8	723.3809	723.3803	723[C_34_H_59_O_16_]^−^	−0.6	Unidentified galactolipid		+	MS data

CSID, LMF designate the ChemSpider and LIPID MAPS online database number for the metabolite, respectively. * Identity confirmed with the reference. ^#^ Abbreviations: ID no., identification number; t_R,_ retention time; MS, mass spectrometry. ^#^ Retention times of organic acids in the analysis of tuber extracts were slightly shifted.

**Table 2:** Effect of *D. caucasica* 70% ethanol extract of leaves on the percentage inhibition of α-glucosidase enzymatic activity.

Conc., µg/mL	With 0.1 M Na_2_CO_3_	Without 0.1 M Na_2_CO_3_	Percentage Difference	*p-*Value
3.125	16.93 ± 1.43	11.94 ± 1.22	−4.990	0.01
6.25	32.73 ± 0.54	26.47 ± 1.43	−6.260	0.0021
12.5	42.81 ± 0.67	39.32 ± 1.75	−3.490	0.0321
25	52.49 ± 0.47	52.31 ± 1.64	−0.180	0.8639
50	67.69 ± 0.26	72.57 ± 1.52	4.880	0.0054
100	59.30 ± 1.1	78.49 ± 2.39	19.19	0.0002

*p*-value = level of significance; the values are the means ± standard deviation (*n* = 3).

**Table 3:** Parameters of α-glucosidase inhibition kinetics.

Inhibitor	Parameters	Enzymatic Reaction I	Enzymatic Reaction II	Mode
None	K*_m_*, mg/L	80.873 ± 0.26	78.55 ± 0.11	
	V*_max_*, mg/L·min	0.597 ± 0.07	1.43 ± 0.183	
	CE	0.007	0.018	
Leaf extract,	K*_m_*_(_*_app_*_)_, mg/L	29.761 ± 1.54	39.984 ± 0.31	
15 µg/mL	V*_max_*_(_*_app_*_)_, mg/L·min	0.157 ± 0.03	0.402 ± 0.041	Mixed-type
	K*_i_*, µg/mL	5.35	5.86	
	CE	0.005	0.01	
Leaf extract,	K*_m_*_(_*_app_*_)_, mg/L	33.615 ± 0.75	86.909 ± 0.16	
25 µg/mL	V*_max_*_(_*_app_*_)_, mg/L·min	0.134 ± 0.004	0.449 ± 0.026	Mixed-type
	K*_i_*, µg/mL	7.23	11.44	
	CE	0.004	0.005	
None	K*_m_*, mg/L	31.446 ± 0.28	-	
	V*_max_*, mg/L·min	1.023 ± 0.23	-	
	CE	0.032		
	K*_m_*_(_*_app_*_)_, mg/L	69.34 ± 0.31	-	
Tuber extract,	V*_max_*_(_*_app_*_)_, mg/L·min	0.991 ± 0.22	-	Competitive
200 µg/mL	K*_i_*, µg/mL	165.97	-	
	CE	0.014	-	
	K*_m_*_(_*_app_*_)_, mg/L	83.125 ± 0.51	-	
Tuber extract,	V*_max_*_(_*_app_*_)_, mg/L·min	1.034 ± 0.38	-	
500 µg/mL	K*_i_*, µg/mL	304.32	-	
	CE	0.011	-	

K*_m_* and V*_max_* in the absence of inhibitor; K*_m_*_(_*_app_*_)_ and V*_max_*_(_*_app_*_)_ are apparent as K*_m_* and V*_max_* in the presence of inhibitors; Enzymatic reaction I: K*_m_*_(_*_app_*_)_ and V*_max_*_(_*_app_*_)_ are apparent as K*_m_* and V*_max_* in the presence of inhibitors (with reagent 0.1 Na_2_CO_3_); Enzymatic reaction II: K*_m_*_(_*_app_*_)_ and V*_max_*_(_*_app_*_)_ are apparent as K*_m_* and V*_max_* in the presence of inhibitors (without reagent 0.1 Na_2_CO_3_; CE is the catalytic efficiency. Values represent mean ± standard deviation of triplicate samples. K*_i_*_,_ inhibition constant of the reactions.

**Table 4:** The effect of *D. caucasica* 70% ethanol leaf extracts on specific activity of AChE (in µM/min/mg protein) and percentage inhibition (in brackets).

No Inhibitor	*D. caucasica*, µg/mL				
-	25	40	50	80	100
0.138 ± 0.001 ^a^ (0)	0.1 ± 0.002 ^b^ (27.55)	0.082 ± 0.002 ^c^ (40.58)	0.078 ± 0.002 ^d^ (43.48)	0.076 ± 0.002 ^e^ (45.31)	0.074 ± 0.002 ^e^ (45.85)

The values with different superscript letters are significantly different, according to Fisher’s least significance difference (LSD) test (*p* < 0.05) after one-way ANOVA.

**Table 5:** Enzyme inhibition patterns [50].

Type of Inhibition	Effect on V*_max_*	Effect on K*_m_*	Effect on Slope of L–B plot	Position of Intersection of L–B plots
Competitive	No change	Increase	Increase	Ordinate axis
Uncompetitive	Decrease	Decrease	No change	None
Non-competitive (a) Simple	Decrease	No change	Increase	Abscissa axis
(b) Mixed: K*_i_* < K*_i_*(A)	Decrease	Increase	Increase	Second quadrant
(c) Mixed: K*_i_* > K*_i_*(A)	Decrease	Decrease	Increase	Third quadrant

Abbreviations: L–B; Lineweaver-Burk plot.

## Data Availability

Not applicable.
